# Femoral lengthening and deformity correction using the Fitbone motorized lengthening nail

**DOI:** 10.1007/s00776-014-0659-3

**Published:** 2014-10-19

**Authors:** Metin Küçükkaya, Özgür Karakoyun, Sami Sökücü, Ramazan Soydan

**Affiliations:** 1 Department of Orthopedics and Traumatology, İstanbul Bilim University, İstanbul, Turkey; 2 Büyükdere Cad. No:120 34394 Esentepe Şişli, İstanbul, Turkey; 3 Department of Orthopedics and Traumatology, Namık Kemal University, Tekirdağ, Turkey; 4 Namık Kemal Üniversitesi Tıp Fakültesi Dekanlığı Namık Kemal Mahallesi Kampüs, Caddesi No:1 Suleymanpasa, Tekirdağ, Turkey; 5 Department of Orthopedics and Traumatology, Metin Sabanci Baltalimani Osteopathic Training and Research Hospital, Istanbul, Turkey; 6 Rumeli Hisarı Caddesi No: 62, 34470 Baltalimanı İstanbul, Turkey

## Abstract

**Background:**

This study reports our results with retrograde Fitbone insertion in patients with femoral shortening and deformity. We also present our experience regarding the benefits, complications, and factors associated with complications of the Fitbone technique.

**Methods:**

Twelve males and ten females had femoral shortening and deformities treated using the retrograde Fitbone technique between 2009 and 2012. The etiologies were post-traumatic in 12 patients, poliomyelitis in four, cosmetic in two, congenital hypoplasia in two, achondroplasia in one, and Perthes sequela in one.

**Results:**

The follow-up time was 30.8 months. The mean lengthening was 5.8 (range 2–14) cm. The degree of acute angular correction was 9° (5–22°) in nine cases. The time to full weight-bearing was 5.9 months. The consolidation index was 1.07 (0.75–1.62) months/cm. Complete consolidation was obtained in all cases except two. Running back was observed in two cases.

**Conclusions:**

The Fitbone technique allows accurate deformity correction. The rigid reamers allow the surgeon to use the Fitbone even in patients with a narrow medullary canal. As this might result in poor bone regeneration, thinner lengthening nails should be considered.

## Introduction

Classical limb-lengthening techniques have disadvantages such as external fixation problems, decreased patient comfort, and rehabilitation difficulties. While lengthening over nail techniques decrease these problems, the major reported complications are related to the pin [1–3].

Motorized lengthening nails have been used for the last two decades [4–9]. Our experience includes fully implantable motorized lengthening nails (Fitbone, Wittenstein; Ingersheim, Germany), intramedullary skeletal kinetic distractors (ISKD; Orthofix; Lewisville, TX, USA), and PRECICE (Ellipse Technologies; Irvine, CA, USA). Here, we report our results of retrograde Fitbone applications in patients with femoral shortening and deformity. We also present our experience regarding the benefits, complications, and factors associated with complications of the motorized lengthening nails technique.

## Patients and Methods

This study included 25 femurs of 22 patients (12 females, 10 males) with leg length discrepancy (LLD) treated using motorized lengthening nails. The mean patient age was 27.2 (range 13–35) years. The etiologies were post-traumatic (malunion in seven, physeal arrest in five), poliomyelitis (four cases), cosmetic (two cases), congenital hypoplasia (two cases), achondroplasia (one case), and Perthes sequela (one case). In one case, hip arthrodesis was performed for septic arthritis that had occurred 18 years earlier. The preoperative LLD was 5.6 (3–14) cm in the unilateral cases.

Three cases had bilateral lengthening (cosmetic and achondroplasia). One of the two cosmetic cases used four Fitbones in the ipsilateral femur and tibia, using the same incisions. In the cosmetic cases, the left side was operated on after the right side had healed completely. The results of tibia applications were not included in this study.

**Fig. 1:**
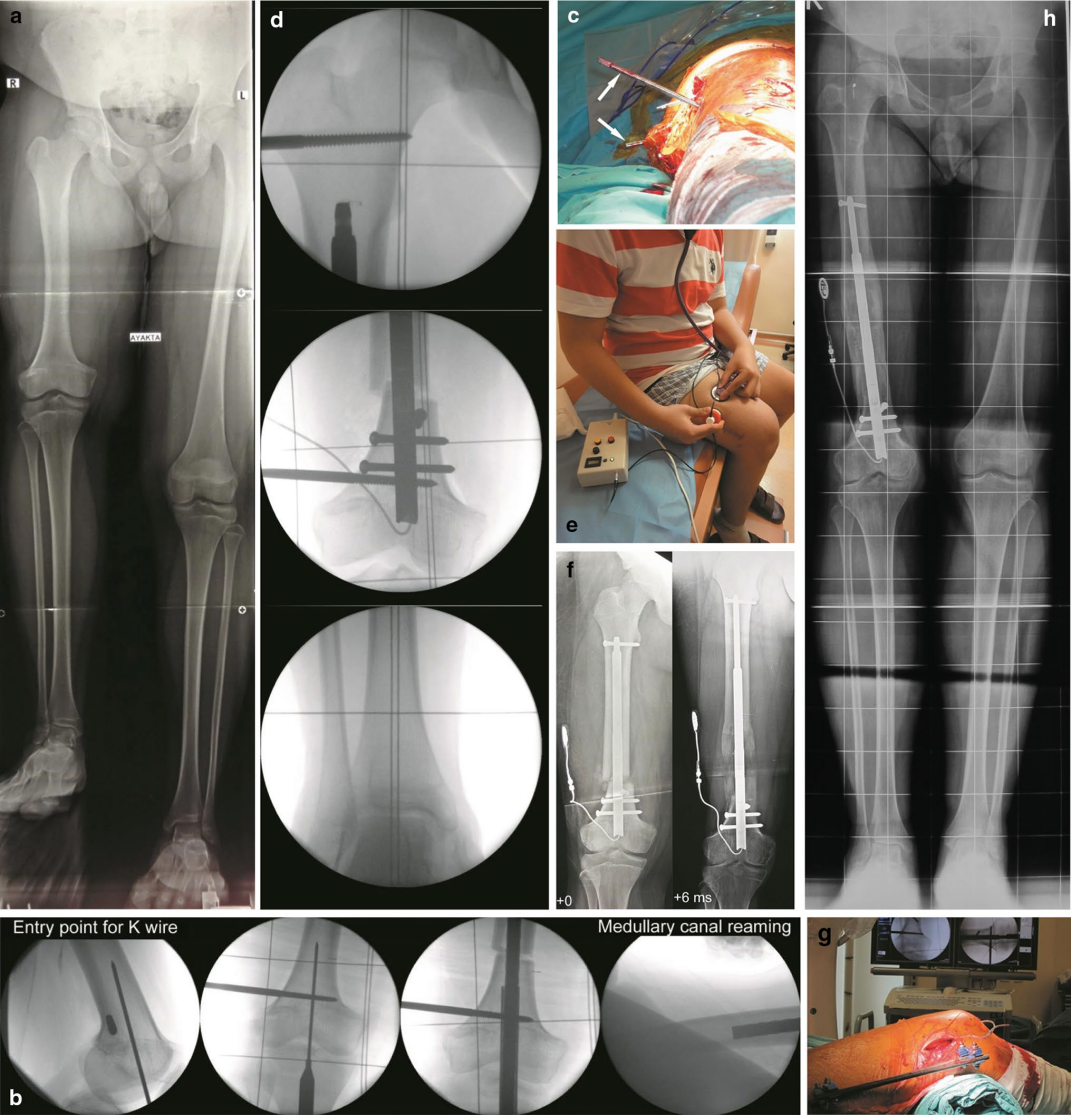
A 14-year-old male with 14 cm of femoral shortening, 6° varus, and 30° rotational deformity was treated with two consecutive retrograde Fitbone applications. a**)** Preoperative LSR while the leg length differences were compensated for using plates and the patellae were oriented anteriorly. b**)** The standard entry point of the K-wire for retrograde femoral nailing and medullary canal reaming was used with rigid reamers. The K-wire should be placed exactly as in the preoperative plan for accurate deformity correction. c**)** A 30° acute rotational correction was completed before reaming the proximal medullary canal. d**)** Position of the blocking screw and intraoperative alignment control using a grid plate (see the text). e**)** Distraction using activation of the transmitter head and the control electronics. f**)** The x-rays in the early postoperative period and 6 months after 8 cm of distraction. g) The Fitbone was replaced with a new one after the first distraction period, and the femur was distracted a further 6 cm. A temporary external fixator was used intraoperatively to protect the distraction site. h**)** LSR after 14 cm of lengthening and deformity correction

Two Fitbones were used consecutively in one patient who had a 14 cm LLD (Fig. 1a–h). In the cases with achondroplasia and congenital hypoplasia, the femur was not long enough to fit a standard femur Fitbone (TAA 24.5 cm, maximum stroke 80 mm). In the achondroplasia case, a shorter custom-made Fitbone (19.5 cm, maximum stroke 60 mm) was used on each side. In the congenital hypoplasia case, a tibia nail (22.5 cm, maximum stroke 60 mm) was used with the retrograde technique for the femur. Acute angular or rotational correction and simultaneous lengthening were performed in nine cases.

The one ipsilateral hip arthrosis and LLD case, total hip replacement, and Fitbone application was performed during the same operation. Another LLD case had ipsilateral femoral and tibial deformities. Femoral deformity was treated with Fitbone and the tibial deformity was treated with a plate in the same session.

### Retrograde Nailing Technique for the Femur

The LLD and angular and rotational deformities were determined from the physical examination and plain radiographs (Fig. 1a). A long-standing radiograph (LSR) was obtained while the leg length differences were compensated for using plates, and the patellae were oriented anteriorly. The malalignment test described in Paley [10] and the reverse planning method in Baumgart [11] were used for preoperative planning. The required deformity correction, entry site, canal diameter, osteotomy site, and positions of the blocking screws were determined on the LSR.

The patient was placed in the supine position on a radiolucent operating table with the knee in a semiflexed position. A radiolucent grid plate was placed under the patient. Before the osteotomy, two parallel 6 mm Schanz pins were inserted into the proximal and distal segments to prevent malrotation after the osteotomy. The distal Schanz pin was inserted first, and it was to be placed posterior to the reamer and nail passage. The second Schanz pin needed to be placed at the level of the lesser trochanter. A Kirschner (*K*)-wire was inserted at the standard entry point for retrograde femoral nailing on the medial part of the intercondylar notch in the anteroposterior view and at the apex of Blumensaat’s line, which is approximately 1 cm anterior to the posterior cruciate ligament in the lateral view. The *K*-wire should be placed exactly as in the preoperative plan (Fig. 1b). Then, the rigid entry reamer was inserted over the *K*-wire to open the cortex, and the medullary canal of the distal segment was reamed with rigid reamers. Use of the tube system for reamers protected the knee joint from reaming debris. Next, an osteotomy was made at the site in the preoperative plan using multiple drill holes. Sufficient drill holes must be created before the osteotomy is completed using an osteotome to prevent medial cortical fracture at the osteotomy site, which would compromise the distal locking nail. Next, the guidewire was inserted in the proximal segment, and the medullary canal was reamed with rigid reamers. Reaming of the dense posterior cortical wall was completed meticulously (Fig. 1b). If rotational correction was necessary, it was to be performed before reaming the proximal segment (Fig. 1c). The medullary canal was over-reamed 0.5 mm larger than the nail (the larger part of the nail was 12 mm). Further over-reaming and high-speed reaming using a rigid reamer could cause necrosis of the bone. The reaming should be done gently to avoid compromising the bone circulation, which could prevent bone regeneration at the distraction site.

Medullary canal preparation was controlled using a nail dummy. The prepared medullary canal must permit smooth, easy insertion of the nail dummy. Next, a tunnel for the cable between the motor unit and subcutaneous antenna was prepared using a drill and the aiming guide.

After the distal metaphyseal osteotomy, the short distal femur fragment is under the influence of deforming muscle forces and the distal metaphyseal bone does not allow the intramedullary nail to have cortical contact. With the lengthening nail, the alignment tends to be displaced or angulated after distraction. If necessary, distal and proximal femur blocking screws should be used in both the distal and proximal segments to prevent malalignment and loss of correction (Fig. 1d).

Then, the Fitbone was inserted carefully without resistance and locked distally and proximally. The cable unit was introduced through the tunnel and connected with a subcutaneous antenna. Before waking the patient, the nail was checked to determine if it was working.

Femoral lengthening using an intramedullary nail occurs along the anatomical axis. It results in coronal plane translation of the knee relative to the mechanical axis. To prevent this malalignment, the distal fragment is translated laterally during the index operation, even if there is no preoperative deformity (Fig. 1d).

Mobilization with two crutches and active knee extension exercises were started the day after surgery. It was vital to arrange active knee joint exercises for the patients until the end of the distraction period. Distraction was started on the fifth postoperative day and performed at 1 mm/per day and in three equal increments (Fig. 1e). Partial weight-bearing (20 kg, plantar contact) was permitted during the distraction period. The patients were followed every 2 weeks with radiographs until the desired distraction length had been achieved. Subsequently, they were seen every 4–6 weeks. The cases with a formation of at least three cortices on anteroposterior and lateral radiograms were considered as having enough consolidation and full weight-bearing was permitted.

## Results

The average follow-up period was 30.8 (range 16–45) months. The mean lengthening was 5.8 (range 2–14) cm. The mean acute angular correction was 9° (5–22°) in nine cases. The time to full weight-bearing (consolidation time) was 5.9 months. The consolidation index was calculated using the interval between the operation and radiographic consolidation date, divided by the distraction length. The consolidation index was 1.07 (0.75–1.62) months/cm. The mean distraction index was 1.1 day/mm, and the mean maturation index was 0.84 (0.42–1.29) month/cm.

Complete consolidation was obtained in all cases except two: one of these cases was a smoker and the other case was the youngest patient (13 years) in this series. In these two cases, healing was obtained after bone grafting. Running back was observed in two cases, owing to a fall in one case and a possible mechanical problem in the other. In these cases, the planned lengthening was obtained after redistraction. At follow-up, a broken proximal locking screw was observed, but it did not cause any problems. During the follow-up period, 14 implants were removed from 12 patients.

The preoperative range of motion was re obtained at the end of treatment in all cases. Scar formation was minimal and all patients approved of the cosmetic results. There was no infection or broken nail in any of our cases.

## Discussion

In recent years, fully implantable lengthening nails have been used increasingly to avoid problems associated with external fixators. The main reported complications related to this nail are motor stop, cable breakage, early or delayed consolidation, subtrochanteric fractures around the proximal locking screw, and joint contractures [9]. Motor stop and cable breakage are usually caused by technical mistakes. The nail can be damaged during insertion if it is hammered or the cable unit can break when it is introduced through the tunnel and connected to the subcutaneous antenna. Another cause of cable unit breakage is inappropriate tunnel preparation, causing friction or crushing the cable during knee movement.

There were two cases of poor bone regeneration in our series. Smoking might explain the insufficient callus formation in one case, while the other case was the youngest patient. We reviewed all of the latter patient’s x-rays and found that the cortical bone was thinner at the end of the distraction period compared to at the beginning. At 10 months, the Fitbone was replaced with a thin standard nail and the distraction site was bone grafted. Solid consolidation was obtained 5 months after bone grafting. We observed that the cortical bone around the nail thickened again after exchanging the nail. We believe that the reason for the poor bone regeneration was excessive reaming of the medullary canal before fitting the nail.

**Fig. 2:**
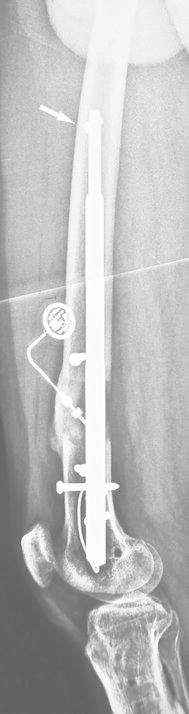
Close positioning of the proximal locking screw weakens the anterior cortical femur, which might increase the risk of fracture

A subtrochanteric fracture around the proximal locking screw has also been reported [9]. This problem might be related to improper reaming technique. Insufficient reaming of the posterior wall of the medullary canal inclines the tip of the sharp rigid reamer anteriorly during reaming. This might cause perforation or weakening of the anterior cortical femur (Fig. 2). We postulate that this is an important cause of subtrochanteric fractures around the proximal locking screw during follow-up.

There are some potential problems related to retrograde femoral nailing, including articular cartilage damage, stiffness, patellofemoral problems, and cruciate ligament disruption. However, prospective studies comparing antegrade and retrograde femoral nailing in terms of these complications have reported similar results for both groups [12]. Retrograde nail insertion does not seem to have a detrimental effect on knee function [13]. We have been using the lengthening over a retrograde nail technique for femoral shortening and deformities and have observed no serious complications related to the knee joint in a small case series [1]. In addition, the tube system used with the Fitbone technique protects the knee from debris or surgical instruments during surgery.

The range of motion is usually good for most of the treatment period using this nail. However, extension loss in the knee, and flexion and abduction contracture in the hip are common problems during femoral lengthening using lengthening nails. These patients usually tend to walk with two crutches without making plantar contact. This walking position causes the contractures around the knee and hip. In addition to active physiotherapy, the use of a long leg brace in the early postoperative period and at night might help to prevent extension loss in the knee. Flexion and abduction contractures in the hip usually resolve spontaneously after full weight-bearing has started.

The consolidation indexes were reported as 1.16 month/cm, 0.86 months/cm, and 0.80 months/cm in studies of lengthening with intramedullary distraction devices [6, 8, 9]. In our study the consolidation index was at a mean of 1.07, which corresponds to similar studies in the literature.

Despite precise preoperative planning, the intended distraction goal might not be achieved in some cases. We believe that the most reliable technique for preventing residual discrepancy is to obtain an accurate LSR at the end of the distraction period. The knee must be in full extension for exact comparison of both sides and the pelvic balance. Therefore, it is extremely important to keep full extension of the knee with active physical therapy until the end of the distraction period.

The motorized lengthening nail has clear advantages for deformity correction and lower limb lengthening. The distraction amount, speed, and rate can be controlled completely and modified according to bone healing, joint contracture, rehabilitation, and pain management. Scar tissue formation is low, and the patient compliance is high. The motorized lengthening nails technique and equipment, such as the rigid reamers and tube system, allow accurate deformity correction. The tube system also allows extraarticular work despite using an intraarticular approach. However, there is no tolerance for technical mistakes. The reverse planning method is crucial for accurate deformity correction, the correct entry point, and the level of the osteotomy. The surgeon must be experienced with medullary canal reaming techniques, blocking screw placement, and with traditional techniques if an unexpected failure occurs during surgery.

The rigid reamer system allows the insertion of a motorized lengthening nail in patients with narrow medullary canals. In these patients, excessive reaming might cause poor bone regeneration; therefore, thinner lengthening nails should be considered.
